# Right Place, Wrong Species: A 20-Year Review of Rabies Virus Cross Species Transmission among Terrestrial Mammals in the United States

**DOI:** 10.1371/journal.pone.0107539

**Published:** 2014-10-08

**Authors:** Ryan M. Wallace, Amy Gilbert, Dennis Slate, Richard Chipman, Amber Singh, Cassie Wedd, Jesse D. Blanton

**Affiliations:** 1 Division of High-Consequence Pathogens and Pathology, Centers for Disease Control and Prevention, Atlanta, Georgia, United States of America; 2 National Wildlife Research Center, United States Department of Agriculture, Fort Collins, Colorado, United States of America; 3 Wildlife Services, United States Department of Agriculture, Concord, New Hampshire, United States of America; University of Pretoria, South Africa

## Abstract

**Introduction:**

In the continental US, four terrestrial mammalian species are reservoirs for seven antigenic rabies virus variants. Cross species transmission (CST) occurs when a rabies virus variant causes disease in non-reservoir species.

**Methods:**

This study analyzed national surveillance data for rabies in terrestrial mammals. The CST rate was defined as: number of rabid non-reservoir animals/number of rabid reservoir animals. CST rates were analyzed for trend. Clusters of high CST rate counties were evaluated using space-time scanning statistics.

**Results:**

The number of counties reporting a raccoon variant CST rate >1.0 increased from 75 in 1992 to 187 in 2011; counties with skunk variant CST rates >1.0 remained unchanged during the same period. As of 2011, for every rabid raccoon reported within the raccoon variant region, there were 0.73 cases of this variant reported in non-reservoir animals. Skunks were the most common non-reservoir animal reported with the raccoon rabies variant. Domestic animals were the most common non-reservoir animal diagnosed with a skunk rabies virus variant (n = 1,601). Cross species transmission rates increased fastest among domestic animals.

**Conclusions:**

Cross species transmission of rabies virus variants into non-reservoir animals increases the risk of human exposures and threatens current advances toward rabies control. Cross species transmission in raccoon rabies enzootic regions increased dramatically during the study period. Pet owners should vaccinate their dogs and cats to ensure against CST, particularly in regions with active foci of rabies circulation. Clusters of high CST activity represent areas for further study to better understand interspecies disease transmission dynamics. Each CST event has the potential to result in a rabies virus adapted for sustained transmission in a new species; therefore further understanding of the dynamics of CST may help in early detection or prevention of the emergence of new terrestrial rabies virus variants.

## Introduction

Rabies has one of the highest known infectious disease case fatality rates, with an estimated 55,000 deaths per year occurring primarily in developing countries where canine rabies has not been controlled [1]. Historically, rabies was responsible for hundreds of human deaths each year in the US, prior to the development of laws promoting responsible pet ownership, canine rabies vaccination campaigns, and increasing availability of effective rabies post-exposure prophylaxis (PEP) [2]. By 1970 the majority of human rabies cases resulted from exposures to rabid wildlife rather than dogs. In paradoxical fashion, by the mid -1970's, when the rabies burden in the US was at presumptive post-European colonization lows, one of the largest recorded wildlife disease epizootics was beginning in rural West Virginia [2], [3].

In 1977, translocation of raccoons from Florida to West Virginia for hunting purposes ignited a rabies epizootic in the local rabies-naïve raccoon populations [4]. Over the next 20 years raccoon variant rabies spread to all mid-Atlantic and northeastern states [2]. In stark comparison to the rabies epidemiology prior to 1970, over 90% of rabies cases are now reported from wildlife species and over 60% of these cases are reported from states that have enzootic circulation of the raccoon rabies virus variant [3], [4]. As evidence from this epizootic event, the introduction of novel rabies virus variants into naïve populations can have longstanding consequences on wildlife populations and human disease prevention. While domestically acquired human rabies deaths are at an historic low and canine rabies virus has been eliminated in the US, the risk of rabies transmission from wild animals remains substantial. More than four million animal bites are estimated to occur in the US each year [7]. Only a fraction of these are reported to public health officials [5], [6]. Despite poor reporting of animal bites, an estimated 35,000 persons (11/100,000 persons) still receive PEP each year due to potential rabies virus exposures [2], [10], [11].

In the continental US, wildlife species in the orders *Carnivora* and *Chiroptera* are responsible for enzootic circulation of rabies virus. These include: foxes (*Vulpes lagopus* and *Urocyon cinereoargenteus*), raccoons (*Procyon lotor*), skunks (*Mephitis spp*), and bats (multiple species) [7]. Seven distinct antigenic rabies virus variants are associated with these four terrestrial species [4]. In addition, more than 10 rabies virus variants are associated with as many species of bats [8]. Each distinct rabies virus variant is maintained within a specific animal reservoir species [9]–[12]. The geographic boundaries of these terrestrial rabies virus variants are dynamic and well described. Nearly all rabid terrestrial mammals are infected with the predicted terrestrial rabies virus variant associated with the geographic region where infection occurred, and very few cases of terrestrial rabies occur in geographic regions with only bat rabies virus variants [10], [13], [14].

Despite the apparent host adaptation and affinity displayed by rabies virus, all mammals are susceptible to this disease. Cross species transmission (CST) occurs when a rabies virus variant adapted to transmission in a specific reservoir animal is transmitted to a non-reservoir animal. Cross species transmission rarely results in successful propagation within a non-reservoir population, referred to as a host shift event [12], [15], [16]. However, the diversity of the seven terrestrial rabies virus variants in the US are apparently due to sustained host shifts from canine rabies virus variants introduced during colonization of the Americas as well as extant chiropteran variants [3]. More recently, repeated CST from bats into skunks and gray foxes in Northern Arizona has resulted in a focal region of sustained bat variant transmission within these terrestrial mammals [11]. Another recent study revealed possible molecular evidence of independent circulation of the California skunk variant in gray foxes following repeated CST events [17]. The impact of these and future potential host shift events are difficult to predict; however, it is possible that such events could impede or compromise current rabies management efforts and jeopardize the health and safety of people and their companion animals. Each instance of CST is an opportunity for viral adaptation resulting in sustained propagation in a non-reservoir species, and CST events are reported in the thousands each year [14]. Currently, public health surveillance is the primary approach for detecting potential host shift events. This study uses routinely collected surveillance data to describe spatiotemporal dynamics of rabies transmission among terrestrial mammals and serve as motivation for new evaluations and the development of techniques to better detect and predict areas at high risk of CST and subsequently where rabies virus host shift events may be more likely to occur.

## Methods

### Data Source

Rabies in animals is a nationally notifiable condition in the U.S. Nearly all animals tested for rabies are submitted due to a bite or other concerning interaction with a human or domestic animal. A small proportion of animals (∼5% annually) are reported through enhanced rabies surveillance involving wildlife that have not exposed humans or domestic animals [14]. National reporting of rabid animals has been conducted since 1938. The CDC program maintains a national database of aggregate reported animal rabies cases by county since 1990. Individual level data including reports for all animals submitted for rabies diagnosis was available since 2006 as described previously [14]. Regional analysis was performed using Health and Human Services regional designations. County-level human population-based rates were calculated from U.S. Census Bureau population data, 2010 census [18].

### Data Selection and Categorization

Reports of all rabid terrestrial mammals within the continental US were compiled from 1990–2011. Rabid bats and humans were excluded from the study, as were data from Alaska, Hawaii, and Puerto Rico. States where only bat rabies virus variants have been reported were also excluded from the analysis (Washington, Oregon, Idaho, Nevada, and Mississippi). Rabies virus variant typing was not available for the majority of reported rabid animals. However, the variant associated with a terrestrial rabies case was assumed based on the geographic location where the animal was reported (e.g. a rabid raccoon in Texas was assumed to be infected with a skunk rabies virus variant). This assumption is based on previously published reports that have found that nearly all rabid terrestrial mammals were infected with the terrestrial rabies variant associated with the geographic region in which the case was reported [10], [13], [14]. This assumption was confirmed by analysis of species and rabies virus variant for reported cases where rabies virus variant information was available (2007–2011). All rabid terrestrial mammals within the defined raccoon variant region were assumed to have the raccoon rabies virus variant [4], [10], [14], [19]. Terrestrial mammals diagnosed with rabies in Arizona and a small region of west Texas were assumed to have either a skunk or fox rabies virus variant, as both variants are present in these regions. Rabid terrestrial mammals in all other counties were assumed to have had one of three skunk rabies virus variants. The three skunk virus variants were analyzed as one group to accommodate instability in predicted geographic boundaries among two adjacent variants. Non-reservoir animals were grouped as raccoons (*P. lotor*), skunks (all species), foxes (all species), domestic animals (cats and dogs), cattle, and other terrestrial mammals (e.g. bobcats, non-cattle ungulates, lagomorphs, rodents, and others).

Counties that reported fewer than three rabid reservoir animals during a 5-year period were excluded from analysis to control for jurisdictions with low surveillance activity. An epizootic of a Mexican canine rabies virus variant occurred in coyotes in southern Texas counties from 1990–1997, therefore rabid coyotes in select southern Texas counties in this time frame were excluded from analysis [20], [21]. In Tennessee, 17 counties were located in a region where the skunk and raccoon variant boundaries overlap. For these counties annual counts of rabid skunks and raccoons were compared and the reservoir was defined by the more frequent of the two potential reservoir species.

### Rate Calculations

The reported rabies CST rate was defined as the rabid non-reservoir animals/rabid reservoir animals. These rates were calculated at the county and HHS regional level, annually and in 5-year aggregate time periods. Annual rates of animal submissions for rabies testing were calculated per 100,000 human population, 2007–2011; the only years when reliable submission data were available. To evaluate the impact of bias, a linear regression model was used to evaluate potential relationship between total submissions of reservoir animals for rabies diagnosis on the CST rate. All comparisons were evaluated at α = 0.05.

### Temporal and Spatial Analysis

Trends in CST rates from 1990–2011 were evaluated using a Monte Carlo permutation model, which detected up to three time intervals where the trend in CST changed significantly [22]. The annual percent change in CST and accompanying 95% confidence interval were reported for the most recent time interval trend identified in the model. Cross species transmission rates were mapped by county by 5-year intervals [23]. A spatial-temporal scan statistic was used to identify county level clusters with a higher than expected CST rate in the raccoon rabies enzootic region from 2007–2011.The scan statistic was run and presented at multiple levels (2, 4, 8, 12, 25, and 50 percent) for the maximum spatial cluster size parameter (MSCS; i.e. the maximum percentage of the population at risk included in a scanned cluster) to increase sensitivity in relation to this parameter setting [24].

## Results

### Annual Trends in Rabies Spillover

#### Raccoon Variant Region

**Figure 1:**
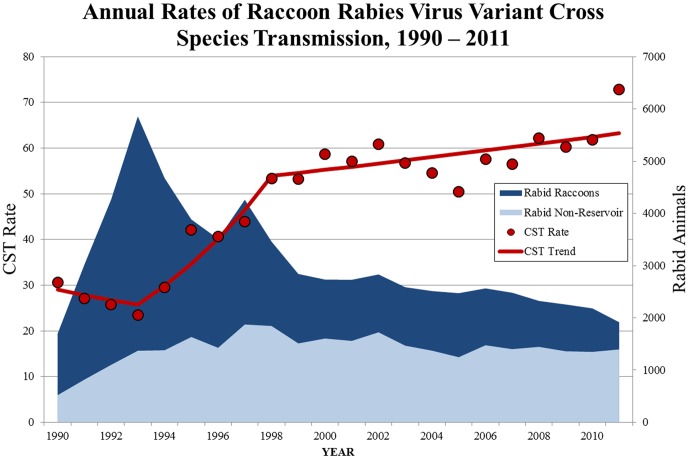
Annual Rates of Raccoon Rabies Virus Variant Cross Species Transmission, 1990–2011.

A total of 67,058 rabid raccoons and 30,876 other animals were reported with raccoon rabies virus variant from 1990–2011. The CST rate ranged from 0.31 in 1990 to a high of 0.73 in 2011, representing a 138% overall increase (Table 1). Three time intervals with distinct CST trends were identified. From 1990–1993, there was no change in the annual CST rate. From 1993–1998, the CST rate increased at 15.9% each year (95% CI: 7.5–25.0), but plateaued again from 1998–2011 (Figure 1). Skunks were the most common non-reservoir animal reported with the raccoon rabies virus variant (n = 16,600). Overall, the annual CST rate of raccoon rabies into skunks increased 118.0% from 1990–2011 (0.16 to 0.35 respectively). However, CST of raccoon rabies into skunks did not change from 1999–2011 (95% CI: −3.2–1.4) (Table 1).

**Table 1:** Trends in rabies cross species transmission (CST) by species or species grouping, 1990–2011.

		Cross Species Transmission	Rabid Animals 1990–2011	1990 CST Rate	2011 CST Rate	Overall CST Change	Latest Trend Time Period	Model APC*	95% CI	P-Value
**Raccoon**	**CST by Animal**	Skunk	16,600	0.16	0.35	118.0%	1999–2011	−0.9%	−3.2, 1.4	0.4
		Fox	6,537	0.05	0.18	251.0%	1996–2011	4.0%	3.0, 5.0	<0.01
		Domestic	5,028	0.06	0.14	139.7%	1990–2011	5.2%	4.5, 5.9	<0.01
		Cattle	644	0.01	0.02	77.8%	1990–2011	4.0%	2.5, 5.7	<0.01
		Other	2,067	0.03	0.04	57.1%	1990–2011	2.7%	1.7, 3.8	<0.01
		***Total***	***30,876***	***0.31***	***0.73***	***138.2%***	***1998*** **–** ***2011***	***1.2%***	***−0.1, 2.6***	***0.06***
**CST by Region**		Northeast	12,542	0.13	0.62	376.2%	1999–2011	−3.9%	−7.2, −0.6	0.03
		Mid-Atlantic	11,129	0.37	0.79	110.5%	1990–2011	2.7%	2.0, 3.5	<0.01
		Southeast	7,205	0.39	0.76	94.9%	1994–2011	5.8%	5.0, 6.6	<0.01
**Skunk**	**CST by Animal**	Raccoon	148	0.01	0.02	183.3%	1990–2011	6.3%	2.4, 10.3	<0.01
		Fox	309	0.02	0.03	55.6%	1998–2011	9.5%	3.5, 15.8	<0.01
		Domestic	1,601	0.10	0.07	−35.9%	1990–2011	−1.0%	−2.5, 0.5	0.19
		Cattle	1,140	0.11	0.02	−83.0%	1990–2011	−5.7%	−7.8, −3.4	<0.01
		Other	580	0.05	0.04	−13.3%	1990–2011	0.50%	−2.3, 3.3	0.73
		***Total***	***3,778***	***0.28***	***0.17***	***−40.3%***	***1990***–***2011***	***−2.0%***	***−3.6, −0.4***	***0.02***
**CST by Region**		Midwest	1,305	0.48	0.20	−59.0%	1990–2011	−1.5%	−3.6, 0.7	0.16
		South	1,212	0.16	0.17	8.8%	2001–2011	7.0%	3.0, 11.1	<0.01
		Southeast	168	0.28	0.18	−36.4%	2001–2011	13.6%	1.9, 26.7	<0.01
		West	1,093	0.22	0.11	−48.8%	1990–2011	1.1%	−0.8, 3.1	0.25

^*^Modeled annual percent change in cross species transmission rates.

#### Skunk Variant Region

**Figure 2:**
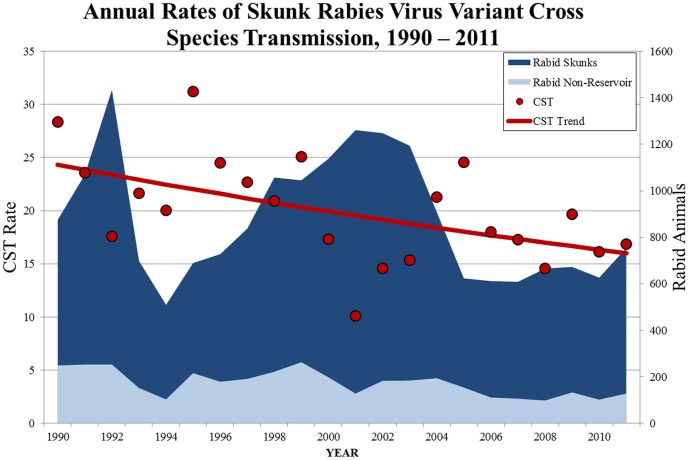
Annual Rates of Skunk Rabies Virus Variant Cross Species Transmission, 1990–2011.

A total of 19,247 rabid skunks and 3,778 other animals were reported from skunk rabies enzootic regions from 1990–2011. Overall, there was a 40.3% decrease in the CST rate of skunk rabies from 1990–2011 (0.28 to 0.17, respectively), with an average decrease of 2.0% (95% CI: −3.6–−0.4) per year (Figure 2).Domestic animals were the most commonly reported non-reservoir animal infected with skunk rabies (n = 1,601) (Table 1). Cross species transmission from skunk to raccoon was a rare event with only 148 occurrences reported from 1990–2011.

### Raccoon and Skunk Variant CST Rates, 2007–2011

Reported CST of raccoon rabies into skunks was 18.7 times more frequent in comparison to skunk rabies transmitted into raccoons (95% CI 14.2–24.5) (Table 2). Transmission of the raccoon variant into foxes was 8.1 times more frequent when compared to skunk rabies virus variants (95% CI 6.3, 10.5). Transmission of the raccoon variant into domestic animals was 1.5 times more frequent than skunk variant transmission to domestic animals (95% CI 1.3, 1.7). In cattle, CST of the raccoon variant occurred less frequently than skunk variants (RR = 0.5, 95% CI 0.4, 0.6). Overall, CST of the raccoon variant was 3.7 times more frequent than skunk variants during the study period (95% CI 3.4, 4.0).

**Table 2:** Animal Submission for Rabies Diagnosis and Cross Species Transmission of Rabies Virus by Non-Reservoir Animal, 2007–2011.

	Raccoon Variant Region			Skunk Variant Region			
	Testing Submissions	Number Rabid (Positive Test Rate)	CST Rate	Testing Submissions	Number Rabid (Positive Test Rate)	CST Rate	Raccoon: Skunk Rate Ratio
Raccoon	53,877	11,134 (20.7%)	-	4,132	78 (1.9%)	0.02	18.7 (14.2, 24.5)
Skunk	10,605	3,379 (31.9%)	0.30	7,485	3,329 (44.5%)	-	
Fox	5,404	1,720 (31.8%)	0.15	698	66 (9.5%)	0.02	8.1 (6.3, 10.5)
Domestic Animals	100,163	1,254 (1.3%)	0.11	36,234	251 (0.7%)	0.08	1.5 (1.3, 1.7)
Cattle	1,923	154 (8.0%)	0.01	1,055	93 (8.8%)	0.03	0.5 (0.4, 0.6)
Other Animals	19,422	436 (2.2%)	0.04	3,411	101 (3.0%)	0.03	1.3 (1.2, 1.4)
*Total*	*191,394*	*18,077 (9.4%)*	*0.62*	*53,015*	*3,918 (7.4%)*	*0.17*	*3.6 (3.4, 3.9)*

### Spatial Analysis

#### Raccoon variant region

**Figure 3:**
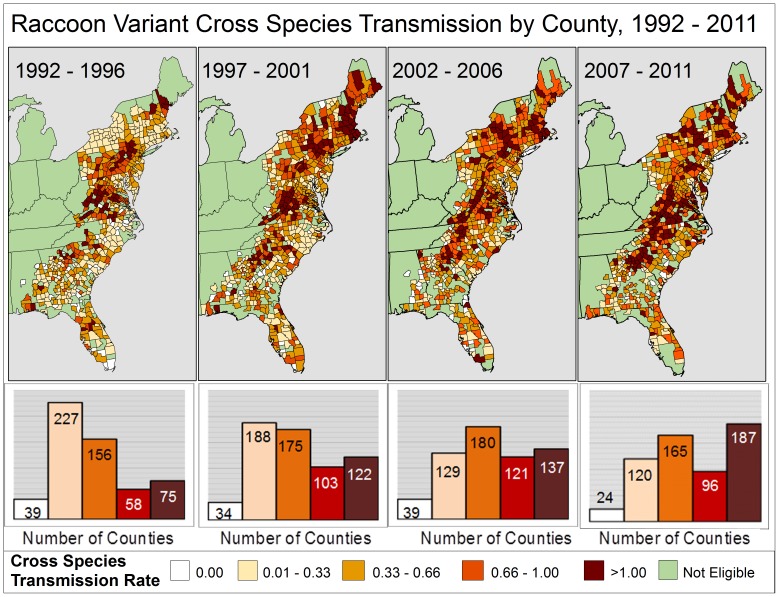
Five-year aggregate spillover rates of raccoon rabies virus variant, by County: 1992–2011.

During 1992–2011, 741 raccoon variant counties met the criteria for inclusion and reported an average of 6.2 rabid terrestrial animals each year. The CST rate doubled during the study period to 0.62 non-reservoir animals diagnosed for every rabid raccoon for time period 2007–2011.During 2007–2011, 187 of 592 (31.6%) counties reported a rate ≥1.0, and increase from 75 counties during 1992–1996 (Figure 3). From 1990–1999, the CST rate trend in the Northeast region increased at 23.8% per year (95% CI 17.1–38.0). This trend changed from 1999–2011, when the CST rate in the Northeast decreased 3.9% per year (95% CI −7.2–−0.6) (Table 1). In its most recent trend time period, 1994–2011, the spillover rate in the Southeast region increased 5.8% per year (95% CI 5.0–6.6). From 1990–2011, the CST rate increased at 2.7% per year in the mid-Atlantic region (95% CI 2.0–3.5).

#### Skunk Variant Region

**Figure 4:**
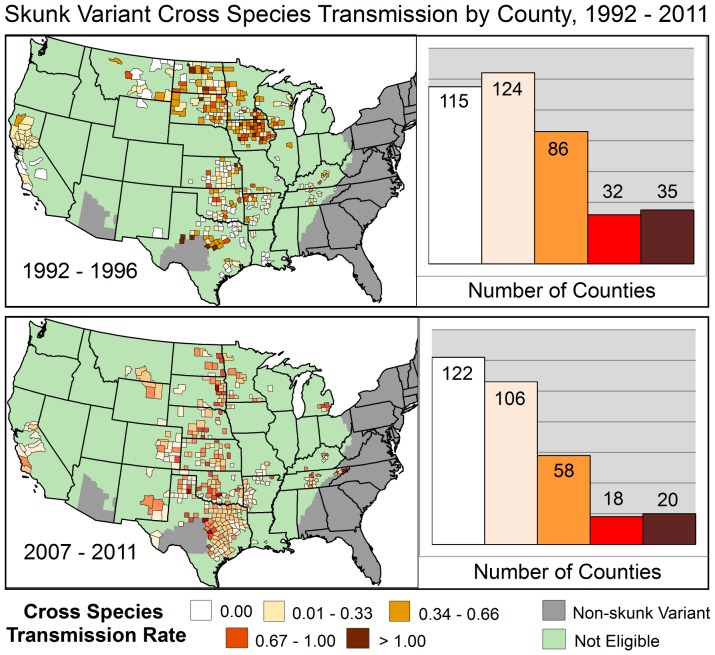
Five-year aggregate spillover rates of skunk rabies virus variants by County: 1992–2011.

During 1992–2011, 754 skunk variant counties met the criteria for inclusion and reported an average of 1.4 rabid terrestrial animals each year. During 1992–1996, 0.22 other terrestrial animals were diagnosed rabid for every reported rabid skunk, and 35 of 392 (8.9%) counties reported a CST rate ≥1.0. During 2007–2011, the CST rate fell to 0.18 and only 20 of 324 (6.2%) counties reported a CST rate ≥1.0 (Figure 4).

In the Southeastern United States, the skunk rabies CST rate trend increased 13.6% per year from 2001–2011 (95% CI 1.9–26.7) (Table 1). In the most recent trend period in the southern United States from 2001–2011, the skunk rabies CST rate increased at 7.0% per year (95% CI 3.0–11.1). No significant changes in the trend or overall CST rate were identified for the Midwest and West regions.

#### Space-Time Cluster Analysis

**Figure 5:**
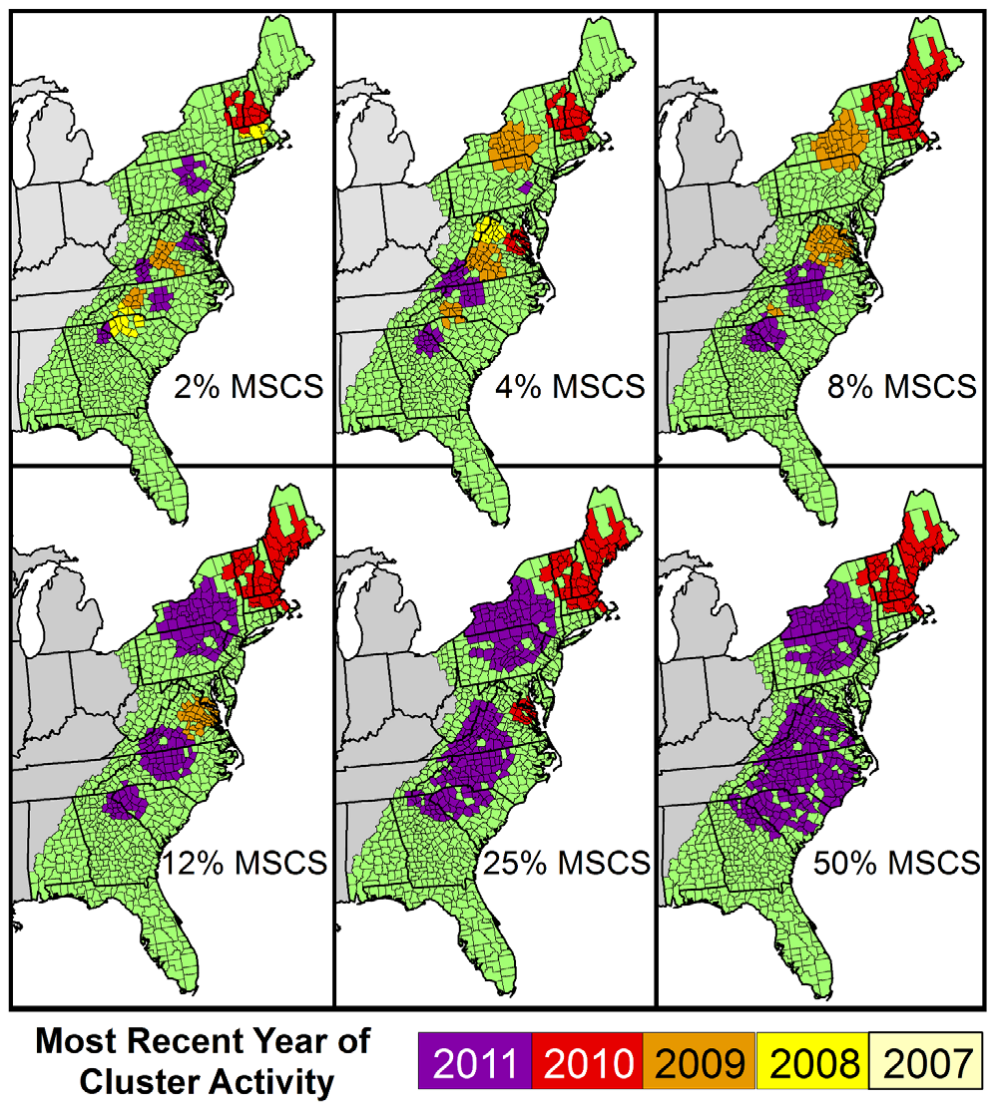
Clusters of High Rates of Raccoon Variant Cross Species Transmission over Varying Spatial Cluster Values, 2007–2011.

Three significant clusters of rabid non-reservoir animals were identified controlling for the number of reported rabid raccoons in the space-time scan statistic at a MSCS of 50% (Figure 5). The two clusters centered on New Hampshire and New York appear to remain fairly stable at lower MSCS levels. However, the risk levels appeared to be heterogenous across the large cluster centered on North Carolina. Additional smaller clusters were identified within this region starting at a MSCS level of 12%.

### Validation of the Variant Assumption

**Figure 6:**
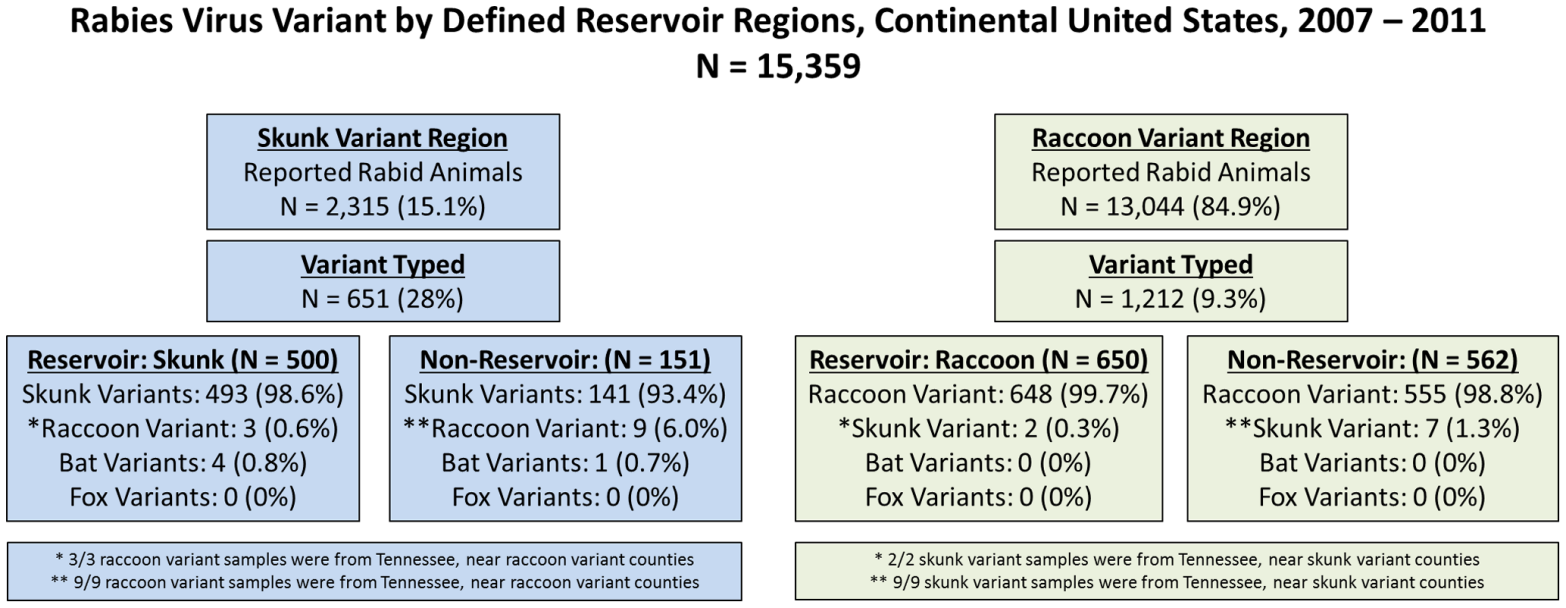
Comparison of Confirmed Rabies Virus Variant and Predicted Rabies Virus Variant, 2007–2011.

Presence of variant typing data were examined for all 15,359 animals analyzed in this study between 2007–2011 (Figure 6). The study-defined raccoon variant region reported 13,044 rabid animals, of which 1,212 (9.3%) were variant typed. Of these 1,212 variant typed animals, 1,203 (99.3%) were infected with the predicted reservoir virus variant. The nine animals which did not have the predicted rabies virus variant were all found to have been infected with a skunk variant. These nine animals were from Tennessee, a state in which both skunk and raccoon rabies virus variants are present. The study-defined skunk variant region reported 2,315 rabid animals, of which 651 (28.1%) were variant typed. Of these 651 variant typed animals, 634 (97.4%) were infected with the predicted reservoir virus variant. Twelve of the 17 animals predicted to have skunk variant rabies virus were actually infected with a raccoon rabies virus variant. All 12 were from the state of Tennessee. The remaining five animals (0.8%) were infected with a bat variant. Of all variant typed animals, 98.6% had the study-defined, predicted rabies virus variant, 1.1% did not have the predicted variant but were from a state in which multiple variants are present, and 0.3% of animals were infected with a non-terrestrial variant.

### Submission of Samples for Rabies Testing

Data on submission of rabies suspect animals for testing were available for 2007–2011. During this time period, 53,877 raccoons and 137,517 other terrestrial animals were submitted for rabies diagnosis in the raccoon variant region (20.7% and 5.0% rabid, respectively). The average annual number of submissions for rabies diagnosis was 57.9 per 100,000 persons residing in the raccoon variant region. From 2007–2011, 7,485 skunks and 45,530 other terrestrial animals were submitted for diagnosis from the skunk variant regions (44.5% and 1.3% rabid, respectively). The average annual number of submissions for rabies diagnosis was 67.9 per 100,000 persons residing in the skunk variant region.

A linear regression model revealed a relationship between CST and submission of raccoons for rabies diagnosis in the raccoon variant region (P = 0.012) (Table 3). However, changes in the number of raccoons submitted had little impact on the CST rate (β = −0.00018). A similar trend was identified in the relationship in the skunk variant region, but with a slightly greater impact on CST rates when the number of skunk submissions is increased or decreased (β = −0.0015, P = 0.008).

**Table 3:** Linear Regression of Reservoir Submission Rate and Cross Species Transmission Rate: Skunk and Raccoon Variant Regions, 2007–2011.

	Variable	DF	Parameter Estimate	Standard Error	t Value	Pr> |t|
Raccoon Variant Region	Raccoon Intercept	1	82.56	3.04608	27.10	<.0001
	Raccoon Submission Rate	1	−0.018	0.00707	−2.52	0.0118
Skunk Variant Regions	Skunk Intercept	1	27.83	2.28046	12.20	<.0001
	Skunk Submission Rate	1	−0.14728	0.05520	−2.67	0.0080

## Discussion

Interspecies rabies transmission can be important in the process of virus adaptation and perpetuation in new hosts [3]. However, from a public health perspective, the more urgent concern is the role CST plays in potentially increasing the threat of rabies virus exposure to humans and domestic animals. The burden and frequency of CST appears to have increased in recent decades and was dramatically higher where the raccoon rabies virus variant is enzootic. This study provides epidemiologic evidence that the raccoon rabies virus variant may be more adept at infecting skunks than previously expected. Whether this is due to viral characteristics or host-host interactions requires further study. Furthermore, this study provides a unique analytic method for the prediction of rabies virus variant host shift events. Routine implementation of this analytic method to annual or real time data could be considered for incorporation into rabies surveillance programs.

### Changing CST Trends

The rate of rabies virus transmission to non-reservoir animals was dynamic over the study period. Perhaps the most dramatic trend observed was the increase in CST of the raccoon rabies variant in the northeastern United States. This increase is most likely explained by the epizootic spread of this rabies virus variant into a previously rabies naïve region. The raccoon rabies variant did not expand into the Northeastern-Atlantic states until the mid-1980's, largely reaching its full northern distribution by 2000 [25]. Prior to the movement of rabies into this region, the raccoon population was rabies-naïve and populations had not experienced decimation due to the epizootic. During the epizootic, interspecies contact with the aberrantly high number of rabid raccoons likely accounts for a sizeable portion of the increasing CST rates observed in these northeastern states. In comparison, the second largest increasing trend in the CST rate was observed in Mid-Atlantic states where the raccoon rabies variant had been established for nearly two decades, and the smallest increase was found in Southeastern states where the variant has been established since the 1940's [26]. In contrast, the three skunk rabies virus variants have been established in the United States since at least the early 1800's; all three variants showed a decline in CST rates [27].

The apparent relationship between the CST rate and length of enzootic status, suggests that the increases in CST in the mid-Atlantic and Northeastern states might be expected to stabilize over time. However, interpretation of this apparent association is difficult, as many potential confounding factors are not collected during routine rabies surveillance efforts. Regional differences in animal population densities and species diversity have the potential to alter disease transmission dynamics. Therefore, ecological studies focusing on inter and intra-species contact rates may help explain the differences in the observed CST trends. Climate change, specifically drought, may also affect CST rates; regions experiencing drought may drive animal populations together as water sources become scarce. Likewise, changes in land use characteristics, such as deforestation or human encroachment into wildlife habitats may also result in changes in animal behaviors. Studies focusing on changing environmental features may help to explain regional differences in rabies transmission dynamics. This analysis describes general trends observed over the past 20 years and is meant to serve as a guide for more focused studies, as described above, to aid in the control of rabies in wildlife reservoir species.

### Raccoons: Super Spreaders?

Raccoons were four times more likely to transmit rabies to other species when compared to skunks. All animal groups, with the exception of cattle, were more likely to be infected with the raccoon variant. Host and environmental factors which facilitate CST are largely unexplored. However, certain behavioral and anthropogenic factors are well described in skunks and raccoons which may explain some of the differences observed in the CST rates of these two species.

Transmission of diseases such as rabies that require direct contact are heavily influenced by the density of susceptible animals and their contact rate with infected animals [28]. Raccoon studies have recorded population densities of 1–250/km2, with higher densities occurring in urban settings [29]–[35]. These studies have also found frequent interspecies interactions. Fewer studies have examined skunk populations, but generally have reported lower population densities of only 3–6/km2 and discrepant reports of the impact of urban environments on population dynamics [36].

Factors other than population density, such as predation or interspecies interactions at communal resources, play integral roles in disease transmission dynamics [37]. Inquisitive species, such as raccoons, and territorial species, such as foxes, may be more likely to physically confront a rabid animal, resulting in increased disease transmission [36], [38]. Raccoons are opportunistic omnivores and prefer den sites near bodies of water and often prosper in high food resource areas such as suburban environment where human refuse, pet food, and other sources of reliable sustenance are likely to be abundant. In these settings, congregations of multiple species at feeding sites are commonplace [39]. Raccoons also commonly utilize latrine sites, which may facilitate interspecies disease transmission, as studies have found that up to 14 different mammalian species may frequent these sites [40]. Skunks, in contrast, typically have lower population densities and are considered an aposematic species; pelage and chemical spray are used as a warning to other species to stay away, which may account for reduced interspecies interactions and thereby lower levels of CST [41], [42]. Aposematic species may be accustomed to being avoided, rather than relying on fight or flight, and therefore less likely to flee when a rabid animal approaches. Skunks also frequently share den sites with raccoons [43]. These ecological and behavioral traits may play a role in their high rate of raccoon variant CST. However, interpretation of these interactions are complicated when the animal in question has rabies, as these animals no longer conform to the behavioral customs attributed to the species [12].

Cross species transmission of rabies among terrestrial animals is a complex interaction that likely depends on animal susceptibility to the virus, animal population densities, animal behavior, niche overlap, landscape characteristics, human population distribution, environmental conditions, and other factors. These multivariate associations can be difficult to evaluate over large geographic areas, even within a single virus variant region. Ecological studies of interspecies rabies transmission should focus on the geographic clusters of high CST rates to maximize detection of CST-related factors. Spatial models should be developed to evaluate the association of these factors with CST rates and identify significant factors that might provide insights which could be used to better understand local epidemiology of rabies and possibly targeted for interventions.

### One Species, One Variant?

Classically, rabies virus variants have been associated with a single species which maintains the variant in nature by conspecific transmission. Cross species transmission of the raccoon variant was unexpectedly high, and the most commonly reported non-reservoir species were skunks. There are multiple theories for increased transmission of raccoon variant into skunks including aforementioned behavioral and ecological patterns that would cause increased interactions between the species [36], [37], [39]–[44]. However, it is not well described as to why these differences would result in increased transmission from raccoon to skunk, but not the inverse. There are potential virus virulence and host susceptibility differences that could affect transmission and result in increased transmission in only one direction, although these are not well studied either [38], [44]–[46].

One potential theory for the unusually high rate of raccoon variant rabies diagnosed in skunks is that this virus has already undergone a host shift into the skunk population. This would artificially inflate the raccoon variant CST rates observed in this study [12], [16]. Propagation of new rabies virus variants occur, presumably on a rare basis, when a virus adapts to a new species resulting in a host-shift and independent circulation in a new species. Over time the virus can be recognized as an independent variant through molecular or antigenic characterization. Molecular analysis of recent host shift events from bats to local mesocarnivores (skunks and foxes) suggested that the rabies virus in those events may have been genetically competent for a host shift prior to transmission (pre-shift adaptation) rather than undergoing genetic adaptation in the new host [11]. Contact rates between bats and mesocarnivores are relatively low, suggesting that transmission of the virus was likely restricted in one direction (i.e. bat to mesocarnivore). However, where contact rates in both directions between two species are relatively high (e.g. skunks and raccoons), it would seem plausible that a pre-shift adapted virus may allow for routine bi-directional transmission between species as well as conspecific circulation. Maintenance of the rabies virus variant between two hosts may prevent selective pressure on the virus complicating detection of a distinct rabies virus variant associated with a specific species. This theory would need to be tested, likely using both mathematical modeling and deep sequencing of viruses from raccoons and skunks. A comparison of high CST regions to low CST regions within the raccoon rabies virus territory might be an effective comparison for evaluating molecular differences in the rabies virus.

### Limitations: Calculating CST Rates from Passive Surveillance

Nationally, rabies virus variant typing is prioritized among non-reservoir animals or locations where there is increased concern for new variant introduction. Variant typing is an added cost and burden for public health programs, and has no direct treatment impact for the human or domestic animal that was exposed. Therefore, rabies virus variant typing is performed on only a subset of samples, nationally. A critical assumption of this study was that all terrestrial animals were infected with the predicted rabies virus variant based on geographic epidemiology. The validity of this assumption is supported by the finding that nearly all variant typed animals were infected with the predicted study-defined variant, as has been reported in the literature [10], [13], [14] (Figure 6).

Public health testing of animals for rabies is directly related to the animal's probability of interacting with people or their companion animals. Differing state and local health department policies may also impact and bias animal submissions. Raccoons are often found at high population densities close to human habitats, which could potentially account for higher rates of submission for rabies testing [29]–[33], [47]–[49]. Contrary to this reasoning, our study found that on a per-capita basis, animal submission rates were similar between raccoon and skunk regions. Simple linear regression analysis indicated that the number of submissions of reservoir animals (skunk or raccoon) has little impact on CST rate. While there does appear to be some bias introduced due to surveillance activity it did not appear to be differential based on rabies virus variant and was of low magnitude, suggesting that submission and testing bias may not have a significant impact CST rates.

### Conclusion

Rabies virus host shift events could threaten the rabies prevention success achieved in the past several decades in the United States. Traditionally, host shift events have been detected through astute local health programs. Given the consequences of delayed host shift recognition, new surveillance methods must be developed to rapidly identify potential high risk areas. This study provides two such methods for further exploration; monitoring of CST trends and evaluation of high CST areas. The development of the unique CST analysis has provided epidemiologic support to the theory of independent skunk to skunk transmission of the raccoon rabies virus variant. The development of algorithms to analyze areas at high risk for CST could be incorporated into standard reporting systems to raise awareness for host shift events among relevant health departments, thereby improving early detection of such events. Dogs and cats may have frequent opportunities for encounters with rabid animals and remain a critical barrier to human rabies exposure. The finding that CST rates increased in cats and dogs is a reminder of the importance of maintaining current vaccination status to protect animal health and prevent human exposure. Currently available animal rabies vaccines are effective against all known rabies virus variants in North America. The higher CST rate in the raccoon variant region highlights the burden of rabies in this region and increased risk even when encountering non-reservoir species. Each CST event has the potential to result in the establishment of a new reservoir species; therefore advancing our understanding of the dynamics of CST may help to prevent the emergence of new terrestrial rabies reservoir species.
